# Cost effectiveness of patient education for the prevention of falls in hospital: economic evaluation from a randomized controlled trial

**DOI:** 10.1186/1741-7015-11-135

**Published:** 2013-05-22

**Authors:** Terry P Haines, Anne-Marie Hill, Keith D Hill, Sandra G Brauer, Tammy Hoffmann, Christopher Etherton-Beer, Steven M McPhail

**Affiliations:** 1 Allied Health Research Unit, Southern Health, Corner of Warrigal and Kingston Roads, Cheltenham, Victoria 3192, Australia; 2 Physiotherapy Department, School of Primary Health Care, Monash University, McMahons Road, Frankston, Victoria 3199, Australia; 3 School of Physiotherapy, The University of Notre Dame Australia, Mouat Street, Fremantle, Western Australia 6160, Australia; 4 School of Physiotherapy, Curtin University, Kent St, Bentley, Western Australia 6102, Australia; 5 School of Health and Rehabilitation Sciences, The University of Queensland, Services Road, St Lucia, Queensland 4072, Australia; 6 Centre for Research in Evidence-Based Practice, Bond University, University Drive, Robina, Queensland 4226, Australia; 7 WA Centre for Health & Ageing, Centre for Medical Research and School of Medicine & Pharmacology, University of Western Australia, Stirling Highway, Crawley, Western Australia 6009, Australia; 8 Centre for Functioning and Health Research, Metro South Health, Cnr of Ipswich Road and Cornwall Street, Buranda, Brisbane, Queensland 4102, Australia; 9 Institute of Health and Biomedical Innovation and School of Public Health and Social Work, Queensland University of Technology, Victoria Park Road, Kelvin Grove, Brisbane, Queensland 4059, Australia

**Keywords:** Accidental falls, Cost effectiveness, Economic evaluation, Hospital, Prevention

## Abstract

**Background:**

Falls are one of the most frequently occurring adverse events that impact upon the recovery of older hospital inpatients. Falls can threaten both immediate and longer-term health and independence. There is need to identify cost-effective means for preventing falls in hospitals. Hospital-based falls prevention interventions tested in randomized trials have not yet been subjected to economic evaluation.

**Methods:**

Incremental cost-effectiveness analysis was undertaken from the health service provider perspective, over the period of hospitalization (time horizon) using the Australian Dollar (A$) at 2008 values. Analyses were based on data from a randomized trial among n = 1,206 acute and rehabilitation inpatients. Decision tree modeling with three-way sensitivity analyses were conducted using burden of disease estimates developed from trial data and previous research. The intervention was a multimedia patient education program provided with trained health professional follow-up shown to reduce falls among cognitively intact hospital patients.

**Results:**

The short-term cost to a health service of one cognitively intact patient being a faller could be as high as A$14,591 (2008). The education program cost A$526 (2008) to prevent one cognitively intact patient becoming a faller and A$294 (2008) to prevent one fall based on primary trial data. These estimates were unstable due to high variability in the hospital costs accrued by individual patients involved in the trial. There was a 52% probability the complete program was both more effective and less costly (from the health service perspective) than providing usual care alone. Decision tree modeling sensitivity analyses identified that when provided in real life contexts, the program would be both more effective in preventing falls among cognitively intact inpatients and cost saving where the proportion of these patients who would otherwise fall under usual care conditions is at least 4.0%.

**Conclusions:**

This economic evaluation was designed to assist health care providers decide in what circumstances this intervention should be provided. If the proportion of cognitively intact patients falling on a ward under usual care conditions is 4% or greater, then provision of the complete program in addition to usual care will likely both prevent falls and reduce costs for a health service.

**Trial registration:**

Australia and New Zealand Clinical Trials Register: ACTRN12608000015347.

## Background

Falls are one of the most frequently occurring adverse events that may impact upon the recovery of older hospital inpatients [1]. Reported rates of falls have varied, though rates on acute hospital wards have been lower than those on subacute or rehabilitation wards [2-5]. The consequence of the majority of falls can be considered relatively minor, with approximately two-thirds resulting in no injury [6]. However, falls remain a considerable concern for patients and their family as injurious falls can threaten both the immediate and longer-term health and independence of the individual. There is need to identify cost-effective means for preventing falls to guide appropriate use of limited resources available to prevent falls in hospitals [7].

Several randomized controlled trials have previously been published indicating that falls in hospital can be prevented [4,8-12]. Many of these programs have been targeted, multifactorial intervention programs involving different combinations of individual interventions leaving clinicians and researchers alike puzzling over which specific interventions should be provided on specific wards [13]. Intensive patient education has been a central component in two large trials of successful multifactorial programs for both subacute and acute hospital settings [8,12]. More recently, a large randomized trial investigating two forms of patient education in isolation found that a multimedia patient education program provided with follow-up from a trained health professional reduced falls among cognitively intact hospital patients, and that a less intensive approach of providing multimedia materials only did not reduce falls [11]. Thus, intensive patient education appears to be an efficacious means for preventing falls in the hospital setting.

A key consideration in determining whether an intervention should be provided in a given hospital setting is whether the effects of a program justify the costs of providing that program [7]. To date, no economic evaluations have been published examining the efficiency of these programs using data directly arising from these trials. Only one economic modeling study has been performed focusing on whether a patient education program should be provided to all geriatric rehabilitation inpatients, no geriatric rehabilitation inpatients, or a subgroup of geriatric inpatients selected by hospital staff clinical judgment of being at high risk of falls [14]. This study found that providing intensive patient education to all patients would cost a health service A$1,192 to prevent 3.67 patients from being a faller (that is, experiencing 1 or more falls) during their admission for every 100 patients treated (incremental cost effectiveness = A$325 per faller prevented), whereas providing this intervention only to patients identified as being at high risk by their physiotherapist saved A$2,704 and prevented 2.2 patients from becoming fallers for every 100 patients treated (all costs in Australian Dollars (A$)).

Two assumptions underlying the previous modeling study were that the intervention would be equally effective for all subgroups of patients, and that it would reduce the risk of falls by approximately 30%. These assumptions were based on results of an exploratory subgroup analysis [15] conducted on data from a larger randomized trial, [8] but are now known to not be consistent with the results of the more recent randomized trial [11]. This earlier work was limited also to patients being treated in subacute/geriatric rehabilitation wards, and employed only one cost per faller estimate derived from research in another country and health system.

The present study seeks to examine the efficiency of providing the intensive multimedia patient education program delivered with trained health professional follow-up to cognitively intact hospital inpatients in addition to their usual care in comparison to provision of usual care alone from the health service provider perspective over the inpatient care time horizon [11]. Contrary to the assumptions employed in the economic modeling study described above, the patient education program was found only to be effective for cognitively intact hospital patients where it reduced the rate of falls by more than 50% and the proportion of patients who were fallers by 40%. The present study is the first to model the cost effectiveness of an intervention for the prevention of falls in hospitals using both cost and effect data collected from a randomized controlled trial. This study also sought to measure the economic burden of an in-hospital fall and the cost of a person being a faller.

## Methods

### Design

This study was an economic evaluation (incremental cost-effectiveness analysis) conducted in parallel with a multicenter randomized controlled trial conducted from the health service perspective. The health service perspective was chosen as the health service are the decision makers when determining how to provide care on their wards in relation to falls prevention.

### Participants and setting

Participants in this trial (n = 1,206) were patients over the age of 60 who were admitted to acute (orthopedic, respiratory medicine, general medicine) wards and any patient admitted to subacute (geriatric assessment and rehabilitation, neurological rehabilitation) wards at Princess Alexandra Hospital (Brisbane) and Swan Districts Hospital (Perth), Australia. The Australian health care system contains a mix of both publicly and privately funded health services: both of the hospital sites were public hospital facilities. Health service funding models vary from state to state, between acute and subacute hospital wards and between private and public hospitals. A detailed description of the demographics of study participants has been provided previously [11]. The trial was registered with the Australia New Zealand Clinical.

Trials Registry (ACTRN12608000015347) on 11 January 2008. The investigation was carried out in compliance with the Helsinki Declaration. Ethical clearance was provided by the medical research ethics committee of the University of Queensland and the human research ethics committees of the Princess Alexandra Hospital and Swan Districts Hospital. Participants in the trial provided written informed consent prior to their voluntary participation.

### Intervention

Two patient education models were tested in the randomized controlled trial; provision of multimedia patient education materials in addition to usual care (that is, materials only), and provision of multimedia patient education materials combined with trained health professional follow-up (that is, complete program) in addition to usual care. These were compared to usual care alone. The content of the patient education programs was developed based upon the health-belief model [16,17]. The materials only group was not different to the usual care control in any of the falls outcomes considered and was not considered further in this economic evaluation. A significant group (complete program)-by-cognitive status interaction was identified in the randomized trial where cognitively intact patients who were allocated to the complete program had a lower rate of falls (8.72 vs 4.01 falls per 1,000 patient days, adjusted hazard ratio = 0.43) and a lower odds of patients who became fallers (30 fallers and 280 non-fallers in control group vs 20 fallers and 260 non-fallers in complete program, adjusted odds ratio = 0.51) [11]. Cognitive status was classified according to trial baseline Short Portable Mental Status Questionnaire outcome where scores of 8 out of 10 or above were classified as cognitively intact [18]. Thus, in this economic evaluation, we examined the efficiency of the complete program versus usual care alone among patients who were cognitively intact.

The face-to-face education delivered as a part of the complete program was planned to be delivered across four sessions, however, the education provider had discretion to increase or decrease this number as they saw fit for individual participants. This one-to-one education often took place at the patient bedside, though patients were sometimes moved to private areas to have these discussions. The median (interquartile range) number of minutes of staff time per patient in providing these sessions was 25 (20, 32) minutes in total. Headphones and portable digital video disc (DVD) players were used when interacting with multimedia materials to minimize contamination of control group participants.

### Outcomes

Falls were defined as ‘an event which results in a person coming to rest inadvertently on the ground or floor or other lower level’ [19]. Falls data were collated by a research assistant blind to participant group allocation via three sources: computerized incident reports, hand searching of individual patient medical notes, and weekly face-to-face patient interviews. Falls captured through any of these approaches were included. The blinded research assistant also collated data on the radiological investigations, clinical investigations and treatments (medical, medication and nursing) provided directly as a result of the fall, length of stay and participant admission diagnosis from medical records.

### Valuation of costs

All costs were calculated in A$ using 2008 as a base-year value over the period of a participant’s hospitalization. Costs associated with acute hospitalization (not directly related to falls) following consent to participate in the study were valued using the Victorian Weighted Inlier Equivalent Separation casemix funding system from 2008 to 2009 [20]. At the time of study, this system was the most advanced activity-based funding system in use in Australia. It calculates payments made directly to hospitals for health care services provided by acute hospitals based primarily upon patient diagnosis related grouping and length of stay in hospital. Weighted Inlier Equivalent Separation costs were then multiplied by 1.33 as these payment rates do not cover fixed hospital costs and are recognized as covering only 75% to 80% of total costs for a ward stay [21]. Costs associated with inpatient rehabilitation were calculated using local, site specific per diem cost estimates in use at the time of study (A$805.9 per day in Western Australia, A$879 per day in Queensland).

‘Costs directly related to falls’ were defined as those that could be directly attributed to the fall by specific listing on incident reports or medical records. Costs directly related to falls were collected during the trial by research assistants who were blinded to group allocation. The costs of providing investigations, treatments and subsequent care for people specifically due to a fall were valued for this category of costs. Time spent completing nursing and medical assessments and associated documentation in medical records (if documented as having been completed specifically because of the fall and not as a part of routine ward reviews) were estimated to be 15 minutes per fall for uncomplicated falls, 30 minutes per fall where injuries were noted, and were valued using local wage rates (resident medical officer rate [22], level 1 year 5 nursing officer (equivalent to a nurse with 5 years of experience) pay scale rate [23]) for the relevant staff. These wage rates were inflated (multiplied by 1.3) to account for on-costs (for example, sick leave and annual leave entitlements). Time required to provide additional nursing assessments as a result of the fall (for example, hourly neurological observations for 24 hours) were estimated by consulting with local hospital staff and were valued using the level 1 year 5 nursing officer pay scale. Costs of providing specific radiological investigations were valued using market rates from the private sector. If a patient on a rehabilitation ward fell and injured themselves resulting in an admission to an acute ward not included in the study, then the length of stay and admission diagnosis for that admission was recorded and valued using the Weighted Inlier Equivalent Separation casemix valuation approach as previously described. It was considered important to include these ‘additional’ costs that were directly related to falls in this analysis and in the calculation of the cost per fall estimate as the Weighted Inlier Equivalent Separation (acute wards) and per diem payment systems are not sensitive to the additional workload created by falls beyond additional length of stay at an individual patient level. A total cost variable was calculated for each participant by summing acute care costs, rehabilitation costs, and costs directly attributable to falls.

There were no missing data to be accounted for in this trial as the medical records from which this data were sourced were available for every case. Data from incident reports and medical records was supplemented by weekly and pre-discharge interviews with participants to capture data regarding each fall that may not have been recorded otherwise. One participant withdrew from the trial after consenting to participate, their data was not included in the analysis due to the revocation of consent to use their data for this purpose.

### Burden of disease

The cost of a fall and of a patient being a faller were estimated using two different assumptions regarding costs not directly related to falls, specifically, whether an increase in the length of hospitalization seen among fallers was due to the fall(s). Patients who fall may stay longer in hospital to treat the injuries they sustain as a direct result of the fall. Thus, the first assumption employed was that the greater length of stay observed among fallers, after adjusting for other factors that might contribute to longer length of stay, are entirely due to the falls observed. Under this assumption, a cohort-style analysis approach was pursued where regression analyses were undertaken using total costs (length of stay costs plus costs directly related to falls) as the dependent variable and faller status (0 = non-faller, 1 = faller: to estimate cost per faller) or number of falls (to estimate cost per fall) as independent variables along with confounders of age, gender, admission diagnosis grouping, whether there was admission to a rehabilitation ward, admission health-related quality of life, and history of falls in the 6 months prior to hospital admission. Three regression analysis approaches were used to estimate this amount: linear regression analysis, linear regression analysis with removal of outlier data points (more than 3 standard deviations higher than the mean), and robust regression which first performs an initial screening based on Cook’s distance >1 to eliminate gross outliers before calculating starting values and then performs Huber iterations followed by bi-weight iterations in calculating regression coefficients (again to minimize the influence of outliers) [24]. However, patients who would otherwise have a longer length of stay in hospital may be more likely to be observed to fall during an admission as a consequence of being observed for a longer period of time. Further, a latent (unobserved) variable may be responsible for both the increased length of stay and occurrence of falls that cannot be adjusted for in the regression analyses described above. Therefore burden of disease estimates that excluded costs associated with greater length of stay in fallers under the assumption that they do not cause an increase in length of stay were also calculated. Here the costs directly related to falls were summed and then divided by the number of fallers (to calculate the cost per faller) or divided by the total number of falls (to calculate the incremental cost per incremental fall).

### Incremental cost effectiveness using randomized controlled trial data

The incremental cost-effectiveness analysis examined the cost per fall prevented and cost per faller prevented of providing the complete education program as opposed to usual care among cognitively intact patients in the randomized trial. Usual care in this trial varied from ward-to-ward and between sites in this trial but consisted of use of a locally developed falls risk screening tool and generic interventions (for example, orienting patients to the ward) for all patients. Multidisciplinary input (for example, medical, nursing, physiotherapy, occupational therapy) was routinely provided on all wards, although therapists such as physiotherapists and occupational therapists provided more intensive input on subacute rehabilitation wards. Falls risk alert items (for example, arm bands) were used for those identified as being at high risk. Physical restraint was not a front-line method for managing patients with agitation and/or confusion at either site. All patients in this trial received usual care, those in the control group received usual care alone and no subjects in the control group received the additional education intervention.

The price of delivery of the education program consisted of both the start-up costs to commence providing the education program (training a staff member: A$440, purchase of 2 × portable DVD players: A$150 each, 8 h of staff member time in training) averaged over the expected lifespan of the equipment and need to train another staff member (500 patients), along with ongoing costs for employment of the health professional who provided the education program. Labor input was counted in minutes spent with the patient recorded during the trial. Labor input and staff time spent in training was valued ‘Health Professional Level 3 Step 5’ hourly wage rate [25] with an additional 30% loading for on-costs. This salary level is equivalent to an allied health professional with 4 years of clinical experience.

The difference in costs between groups was estimated from the adjusted regression coefficient derived from a multiple regression model including covariates of age, gender, admission diagnosis grouping, whether there was admission to a rehabilitation ward, admission health related quality of life, and history of falls in the 6 months prior to hospital admission that was restricted to cognitively intact patients only. The difference in effects was taken from a regression of the difference in the proportion of patients falling in each group (fallers) and the total number of falls in each group (falls) adjusted for admission diagnosis and whether there was admission to a rehabilitation ward. Bootstrap resampling was then used to construct 95% confidence intervals around the incremental cost per fall and cost per faller estimates [26], while the output of the bootstrap resampling was used to construct cost-effectiveness acceptability curves [27] to determine the probability that the intervention program was both more effective and less costly from the health service perspective than usual care.

### Decision tree modeling and sensitivity analyses

Modeling is the process of representing the real world with a series of numbers, and mathematical and statistical relationships. Modeling and trial based economic evaluations are complementary tools when forming policy advice, as trial based evaluations alone are rarely sufficient to guide policy development [28]. From our economic evaluation based on trial data, the proportion of cognitively intact participants who fall during usual care conditions is set at levels observed during the trial. However, a policymaker may wish to know how cost effective the intervention might be if it were delivered to a slightly different set of wards with a lower proportion of patients who fall under usual care (perhaps the patient population are at lower risk of falling or the background usual care practices are more effective at preventing falls). Trial based data cannot answer such a question without conducting another trial on such wards. It also cannot estimate the impact on cost effectiveness if the intervention were to be more or less effective as it was found to be in the trial without further trials [28]. Thus modeling is required to examine the impact of broader implementation of a health intervention than what was undertaken in a trial or trials, and to understand how variability in contexts might affect the cost effectiveness of such an implementation.

**Figure 1:**
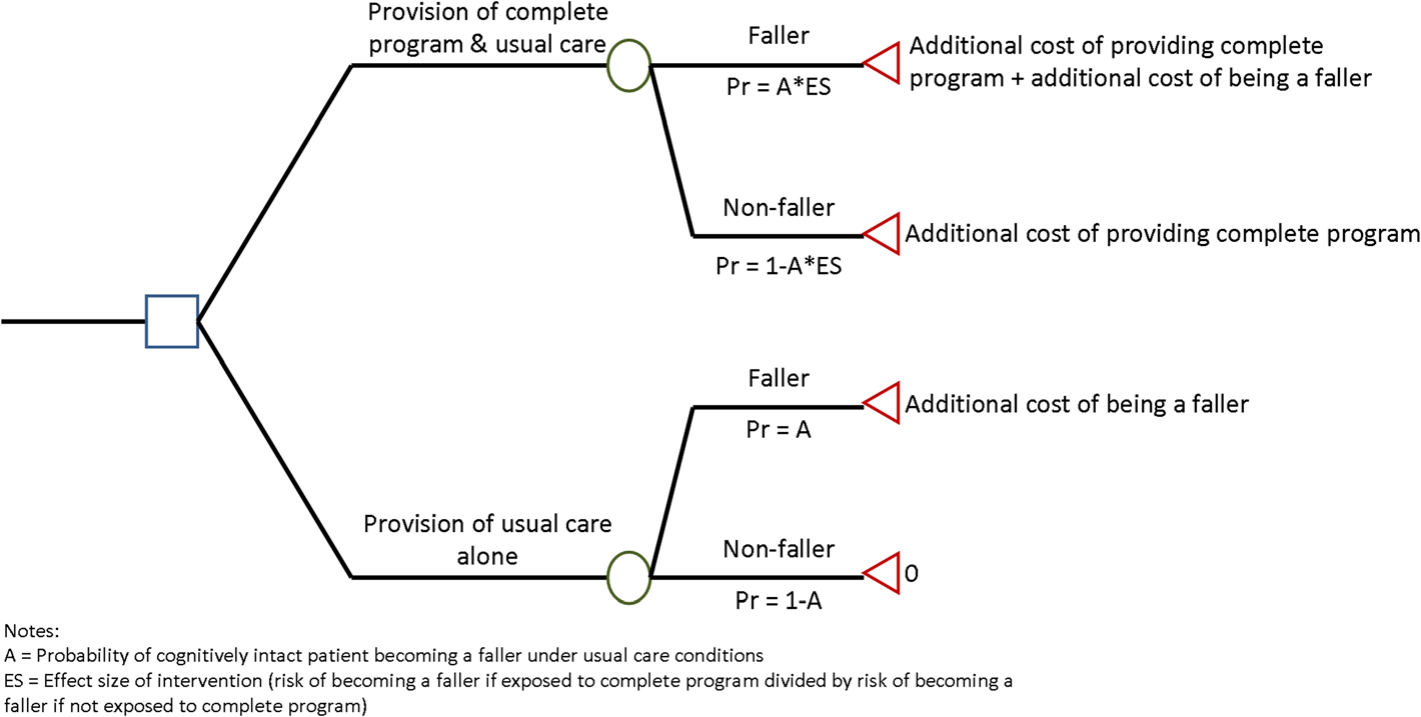
Decision tree structure.

We used a decision tree analysis model to further investigate the incremental cost effectiveness of the complete program compared to usual care alone in preventing fallers and subject it to three-way sensitivity analyses among cognitively intact inpatients (Figure 1). A decision tree model outlines decisions (that is, to provide an intervention or not), the probability or fraction of various outcomes (that is, proportion of patients becoming fallers), and the valuation of each outcome (that is, the cost of a patient becoming a faller). The mean value of a decision is computed analytically by summing the probability of each outcome with its value [29]. The following formula was used: Incremental cost effectiveness (cost per faller prevented) equals:

$$\frac{\left[\mathrm{Cos}t^{\mathrm{intervention}100}+\left(\mathrm{Cost}{\;}^{\mathrm{faller}}*\mathrm{Faller}s^{\mathrm{CP}100}\right)-\left(\mathrm{Cost}{\;}^{\mathrm{faller}}*\mathrm{Faller}s^{\mathrm{UC}100}\right)\right]}{\left(\mathrm{Faller}s^{\mathrm{CP}100}-\mathrm{Faller}s^{\mathrm{UC}100}\right)}$$

Where Cost^intervention100^ = cost of providing intervention to 100 cognitively intact patients; Cost^faller^ = cost of a faller; Fallers^CP100^ = number of fallers among 100 cognitively intact patients under complete program conditions; and Fallers^UC100^ = number of fallers among 100 cognitively intact patients under usual care conditions.

We subjected this decision tree model to three-way sensitivity analyses by varying: (i) the proportion of patients who were cognitively intact who fall on a ward, (ii) the cost to a health service of a patient being a faller, and (iii) the effectiveness of the intervention. The effectiveness of the intervention was modeled as producing a 40% reduction in the proportion of patients who became fallers (taken from trial data), along with more conservative estimates of 30% and 20% reductions. The proportion of patients who were cognitively intact who became fallers on a ward was varied between 3% and 20%. In the main trial, approximately 20% of rehabilitation patients who were cognitively intact fell, and approximately 5% of acute hospital patients who were cognitively intact fell. The cost per faller was modeled using two values taken from the burden of disease analyses in the present study. The first was taken from the costs directly related to falls among cognitively intact patients among all three groups in the study. The second was from the total costs attributable to fallers after adjustment for the other confounders selected, using the robust regression analysis approach (to minimize the influence of outliers and produce a more conservative estimate) restricted to cognitively intact patients. A third cost per faller included in these sensitivity analyses was taken from previous research conducted in the hospital setting [30]. This cost figure was originally expressed as cost per person who incurred one or more injurious falls while in a US hospital but was converted to cost per faller in 2008 A$ using a process previously employed [14]. The cost per injurious faller figure from this previous work was converted to 2008 US$ using the US consumer price index [31], then converted to A$ by using the exchange rate as of 1 September 2008 (study mid-point; A$1 = US$0.8537) [32], then converted to cost per faller by multiplying by the proportion of cognitively intact fallers in the main study who incurred one or more injurious falls defined as falls resulting in bruising, laceration, fracture, loss of consciousness, or patient reports of persistent pain (proportion = 0.49). Threshold sensitivity analyses were also conducted to identify the point at which a policy decision might change (that is, when the intervention both prevents falls and saves resources).

## Results

### Burden of disease

Costs that were counted within this study that were thought to be directly related to a fall (excluding costs associated with length of stay in hospital) were small in comparison to the costs associated with length of stay in hospital (Table 1). For all but one of the subgroups considered, the amount was A$21 or less per patient or A$93 or less per faller. The one exception was the ‘complete program’ cognitively impaired subgroup, which had a mean of A$187 per patient. This was largely driven by one patient who fell and fractured their cervical spine, and was subsequently transferred to an acute ward outside of the study data collection wards specifically for the treatment of this injury. Removal of this participant would have brought this mean figure back to A$19 for this subgroup.

**Table 1:** Breakdown of costs related to acute and rehabilitative care, and costs related to falls for all patients, patients who fell, and patients who had an injurious fall

**Cognitive function classification grouping^a^**	**Control**		**Materials only**		**Complete program**	
	**Intact**	**Impaired**	**Intact**	**Impaired**	**Intact**	**Impaired**
N	280	101	316	108	310	91
Number of falls	46	35	61	35	25	45
Number of fallers	30	24	32	24	20	24
Number of injurious falls	15	10	25	15	10	22
Number of people who had one or more injurious fall	13	8	17	12	10	16
Mean (SD) acute care costs post consent per patient	8,481 (12,856)	5,140 (8,142)	8,927 (16,776)	6,947 (14,079)	10,774 (18,344)	11,128 (28,570)
Mean (SD) rehabilitation costs post consent per patient	10,964 (19,972)	26,050 (36,776)	15,026 (24,925)	24,892 (31,823)	11,197 (18,906)	21,740 (37,130)
Mean (SD) costs of radiological investigations directly related to falls per patient	4 (27)	7 (59)	2 (33)	6 (43)	0 (0)	11 (54)
Mean (SD) medical costs directly related to falls per patient	2 (10)	5 (17)	2 (10)	4 (15)	1 (4)	6 (20)
Mean (SD) nursing costs directly related to falls per patient	1 (4)	2 (7)	1 (5)	8 (63)	0 (2)	5 (15)
Mean (SD) all costs^b^ directly related to falls per patient (excluding acute care and rehabilitation costs)	8 (47)	15 (85)	7 (54)	21 (96)	1 (7)	187 (1,602)^c^
Mean (SD) acute care costs post consent among patients who were fallers post consent	8,556 (13,585)	4,176 (8,130)	11,247 (17,369)	3,000 (5,924)	18,751 (41,564)	5,999 (11,329)
Mean (SD) rehabilitation costs post consent among patients who were fallers post consent	33,317 (29,048)	56,406 (55,296)	45,491 (43,073)	44,959 (45,480)	25,489 (21,284)	53,452 (52,861)
Mean (SD) costs of radiological investigations directly related to falls among patients who were fallers post consent	37 (76)	28 (120)	24 (102)	29 (90)	0 (0)	41 (101)
Mean (SD) medical costs directly related to falls among patients who were fallers post consent	21 (23)	21 (30)	20 (25)	20 (28)	12 (13)	23 (33)
Mean (SD) nursing costs directly related to falls among patients who were fallers post consent	7 (10)	10 (13)	9 (13)	38 (132)	4 (8)	18 (27)
Mean (SD) all costs^b^ directly related to falls among patients who were fallers post consent (excluding acute care and rehabilitation costs)	76 (126)	64 (168)	65 (162)	93 (190)	19 (22)	710 (3,108)^c^
Mean (SD) acute care costs post consent among patients who had an injurious fall post consent	7,811 (14,313)	4,378 (11,513)	5,908 (10,447)	1,848 (3,491)	24,835 (54,923)	8,034 (13,026)
Mean (SD) rehabilitation costs post consent among patients who had an injurious fall post consent	29,700 (21,118)	52,630 (46,211)	40,758 (30,380)	48,853 (40,312)	24,496 (26,482)	51,871 (48,331)
Mean (SD) costs of radiological investigations directly related to falls among patients who had an injurious fall post consent	62 (102)	73 (207)	33 (136)	58 (123)	0 (0)	62 (119)
Mean (SD) medical costs directly related to falls among patients who had an injurious fall post consent	28 (30)	51 (35)	26 (30)	29 (32)	13 (13)	30 (38)
Mean (SD) nursing costs directly related to falls among patients who had an injurious fall post consent	14 (12)	18 (12)	12 (15)	73 (184)	7 (10)	956 (3,722)
Mean (SD) all costs^b^ directly related to falls among patients who had an injurious fall post consent (excluding acute care and rehabilitation costs)	126 (171)	156 (275)	94 (214)	172 (248)	23 (24)	1,058 (3,797)^c^

^a^Based on Short Portable Mental Status Questionnaire cut off of 7/10 or below is impaired.

^b^All costs includes costs of radiological investigations, medical costs, nursing costs, medication costs, on-call payment costs, suture procedure costs, orthoses costs, and other tests costs.

^c^Includes acute care costs of one patient transferred to an acute ward outside of the study following fracture orbital fossa and C2 vertebra fracture as a result of a fall for treatment of this injury.

The cost per fall and cost per faller calculated using the cohort approach are presented (Table 2). Standard regression analyses without removal of outliers identified a cost per fall of A$12,469 and a cost per faller of A$24,927. Re-examining the data in Table 1 reveals that there was little difference between the acute care costs of all patients and fallers, whereas the inpatient rehabilitation costs of fallers were substantially higher than for patients overall. Thus, it appeared that the key driver of the increase in costs observed using the cohort approach was an increased length of stay on rehabilitation wards and not on acute wards. Elimination of outliers 3 standard deviations higher than the mean led to removal of 29 ‘high cost’ participants. Regression analyses with these outliers removed and robust regression analyses both produced similar cost estimates (Table 2), which were approximately one-third less than the estimates modeled using ordinary least squares regression with the outlier variables retained. The cost per faller and cost per fall among cognitively intact patients were similar to those for cognitively impaired patients once the outlier patients were removed from the analyses.

**Table 2:** **Cost per faller (in addition to usual care costs) and cost per fall estimates based on adjusted regression coefficients ((standard error), *P *value) regressing faller (dichotomous) or total number of falls (count) on the overall sum of acute care costs, rehabilitation costs and all costs directly related to falls by subgroup and across all groups**

**Group**	**OLS regression**		**OLS regression with outliers removed**		**Robust regression**	
	**Cost per faller**	**Cost per fall**	**Cost per faller**	**Cost per fall**	**Cost per faller**	**Cost per fall**
Control: cognitively intact	17,240 (3,994), *P* <0.001	8,905 (2,101), *P* <0.001	18,516 (3,322), *P* <0.001	9,695 (1,742), *P* <0.001	21,071 (1,931), *P* <0.001	9,695 (1,743), *P* <0.001
Control: cognitively impaired	35,650 (7,307), *P* <0.001	16,488 (4,041), *P* <0.001	15,664 (4,481), *P* <0.001	14,805 (2,907), *P* <0.001	14,978 (3,932), *P* <0.001	14,804 (2,908), *P* <0.001
Materials only: cognitively intact	17,241 (3,994), *P* <0.001	8,840 (2,040), *P* <0.001	18,322 (3,763), *P* <0.001	7,599 (1,480), *P* <0.001	17,295 (2,310), *P* <0.001	7,599 (1,480), *P* <0.001
Materials only: cognitively impaired	35,650 (7,307), *P* <0.001	6,910 (3,782), *P* = 0.07	719 (6,071), *P* = 0.91	4,901 (2,758), *P* = 0.08	1,566 (6,401), *P* = 0.81	4,901 (2,758), *P* = 0.08
Complete program: cognitively intact	14,301 (5,260), *P* = 0.007	17,571 (3,857), *P* <0.001	5,976 (4,521), *P* = 0.19	7,310 (3,503), *P* = 0.04	6,004 (3,517), *P* = 0.09	7,311 (3,503), *P* = 0.04
Complete program: cognitively impaired	26,843 (11,072), *P* = 0.02	17,178 (4,000), *P* <0.001	20,797 (5,118), *P* <0.001	9,305 (2,217), *P* <0.001	17,156 (3,924), *P* <0.001	1,468 (1,466), *P* <0.001
All cognitively intact patients	21,506 (2,632), *P* <0.001	9,898 (1,326), *P* <0.001	15,759 (2,158), *P* <0.001	8,222 (1,055), *P* <0.001	14,591 (1,431), *P* <0.001	9,273 (704), *P* <0.001
All cognitively impaired patients	26,474 (4,686), *P* <0.001	13,879 (2,088), *P* <0.001	12,274 (2,924), *P* <0.001	8,455 (1,055), *P* <0.001	11,375 (2,534), *P* <0.001	11,074 (1,094), *P* <0.001
All patients	24,927 (2,270), *P* <0.001	12,469 (1,086), *P* <0.001	14,606 (1,679), *P* <0.001	8,454 (839), *P* <0.001	13,522 (1,199), *P* <0.001	9,629 (561), *P* <0.001

Coefficients adjusted for age, gender, admission diagnosis grouping, whether there was admission to a rehabilitation ward, admission health related quality of life (assessed by the EuroQol 5 Dimensions (EQ-5D) instrument), and history of falls in the 6 months prior to hospital admission.

OLS, ordinary least squares.

### Incremental cost effectiveness using randomized controlled trial data

**Figure 2:**
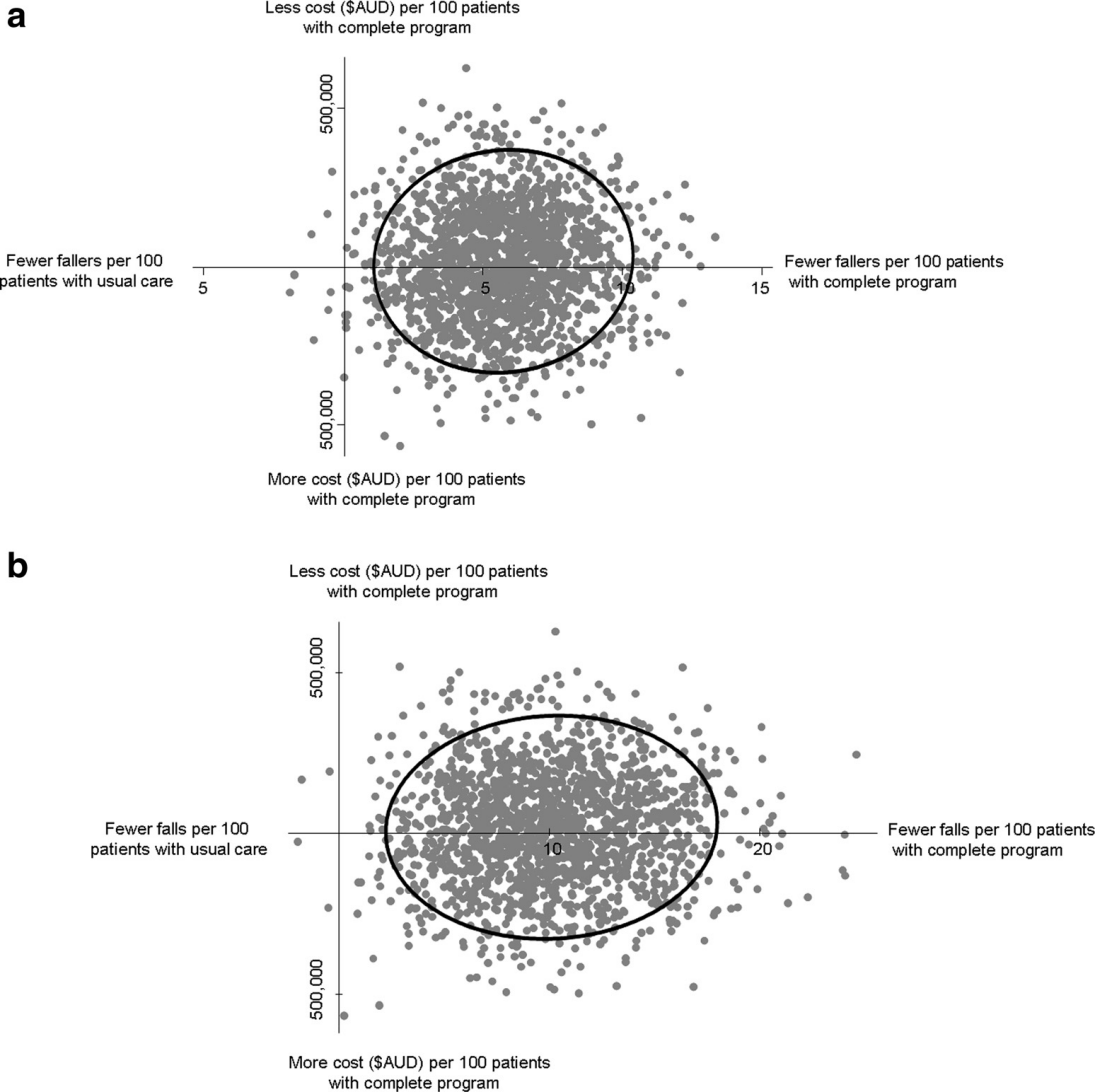
Cost effectiveness of the complete program versus usual care among patients who are cognitively intact with 95% confidence ellipse for fallers (a) and falls (b) prevented.

The incremental cost effectiveness of the complete program relative to usual care was A$526 per faller prevented and A$294 per fall prevented. There was considerable uncertainty surrounding the incremental cost-effectiveness ratios calculated when using primary study data (Figure 2), which was largely driven by outlier patients who had very long lengths of stay. Acceptability curve analysis indicated that stakeholders would need to be willing to pay A$68,108 per faller prevented (in addition to the costs and savings counted in the present analysis) or A$38,213 per fall prevented in order to be 95% confident that the complete program was worthwhile. The probability that the complete program dominated (that is, was both more effective and less costly) the usual care condition for the incremental cost per faller or per fall prevented was 0.52.

### Decision tree modeling and sensitivity analyses

Decision tree modeling of the cost effectiveness of this program and three-way sensitivity analyses used the cost per faller values of A$58 (cognitively intact inpatients across all groups; costs directly related to falls), A$14,591 (cognitively intact inpatients across all groups; total costs associated with being a faller after adjustment for confounders using robust regression), and A$2,867 (translated into A$ at 2008 values from previously published data [30]).

**Figure 3:**
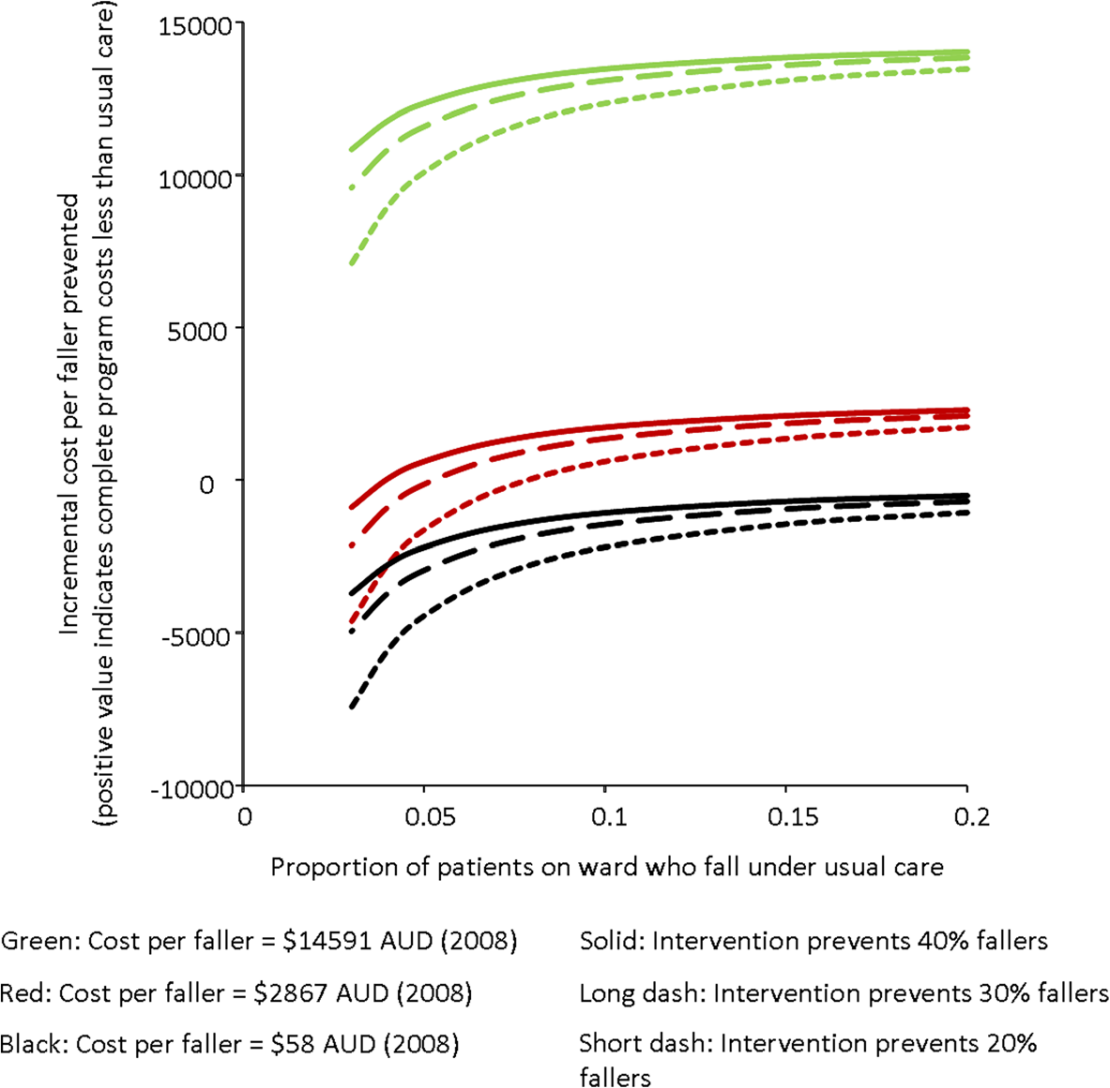
Decision tree modeling with three-way sensitivity analyses of incremental cost effectiveness per faller prevented.

Sensitivity analyses (Figure 3) indicated that the greatest uncertainty in the cost-effectiveness estimates calculated lie in the cost per faller estimates employed. Where the middle cost per faller estimate of A$2,867 was used along with the effectiveness of the intervention program estimate taken from the randomized trial (40% reduction), the complete program appeared to both prevent more falls and cost less than usual care alone from the health service perspective as long as at least 4.0% of cognitively intact patients on a ward were fallers under usual care conditions during their admission. If more conservative estimates of intervention effectiveness were used, then a higher proportion of patients falling under usual care conditions was required (5.3% if the intervention reduced fallers by 30%, 8.0% if the intervention reduced fallers by only 20%). The intervention program did not demonstrate lower costs than usual care in any scenario modeled when the lower cost per faller estimate of A$58 was used. The intervention program demonstrated lower costs than usual care in every scenario modeled when the higher cost per faller estimate of A$14,591 was used.

## Discussion

The present economic evaluation was designed to assist health care providers decide in what circumstances provision of the patient education program should be provided. If the proportion of cognitively intact patients falling on a ward under usual care conditions is 4% or greater, then provision of the complete program in addition to usual care will likely both prevent falls and reduce costs for a health service. Three key caveats should be noted in this recommendation. First, this recommendation is sensitive to the different values of the cost per faller modeled. These estimates were only developed from the health service perspective and were limited to the period of inpatient hospitalization. Costs of care post hospitalization and non-health costs (including legal costs) would add to these estimates. Second, the effectiveness of the program has been derived from a randomized trial conducted across two hospitals in Australia and is relative to the standard of usual care provided in these settings [11]. It is plausible that usual care provided in these sites may be different to other hospitals around the world. Both of these hospitals had falls prevention interventions in place as a part of usual care including use of falls risk screening/assessment tools, provision of falls risk alert signage, nursing falls care plans and provision of multiple allied health therapies including physiotherapy. However, the principle illustrated in our analyses that the cost effectiveness of an in-hospital falls prevention intervention depends heavily on the rate of falls under usual care conditions would still transfer beyond the Australian context. Third, this recommendation assumes no further ‘falls risk screening’ or selective targeting of this intervention to those at higher risk of falls within the targeted population. Previous research indicates that this may lead to greater economic efficiency in delivery of falls prevention interventions [14].

The ‘cost per fall’ and ‘cost per faller’ figures developed using the cohort approach in the present study differed substantially due to different assumptions regarding the effect of falls on length of stay. A recent review of cost per faller estimates from the community and residential care settings have been found to be in excess of US$3,766 (2006 dollar value) and up to US$25,955 in some populations [33] though some concerns with the approaches used have been raised [34]. This indicates that the higher value calculated in the present study may be more accurate, though the authors feel that a value between these extremes is plausible as it is likely that falls may cause some increase in length of stay, but not all of that observed. The value extrapolated from previous work did sit within this range and thus formed the basis for the recommendation provided, even though it was closer to the lower cost per faller estimate than the higher. This value, through the modeling and sensitivity analyses, also generated the closest approximation to the cost-effectiveness ratios seen from the primary randomized trial data.

This study was limited in the precision with which it could construct incremental cost-effectiveness ratios directly from the primary randomized controlled trial data. Collecting cost data concurrently with a randomized trial permits efficiency in data collection, though can often lead to imprecise estimates as ‘total’ healthcare cost data are frequently highly skewed, may contain influential outliers that may be driven by factors other than falls and trials are rarely powered sufficiently to detect differences in costs between groups that would be clinically meaningful. This evaluation was also limited in terms of the perspective taken, being the health service provider, which ignored costs borne by patients and family members (including increased informal care) that might manifest following hospitalization.

The evidence base surrounding the efficacy and now cost effectiveness of intensive patient education programs in the hospital setting is growing. Further work is still required to examine means for successfully incorporating this approach into clinical practice. The present study has examined the cost effectiveness of this approach and provided a conservative guide as to the rate of falls on wards upon which this approach can be implemented to reduce falls and save resources.

## Conclusions

The present economic evaluation was designed to assist health care providers decide in what circumstances provision of this falls prevention intervention should be provided. Conservative modeling from this investigation indicated that if the proportion of cognitively intact patients falling on a ward under usual care conditions is 4% or greater, then provision of the complete program in addition to usual care will likely both prevent falls and reduce costs for a health service.

## Abbreviations

Cost^faller^: Cost of a faller; Cost^intervention100^: Cost of providing intervention to 100 cognitively intact patients; DVD: Digital video disc; Fallers^CP100^: Number of fallers among 100 cognitively intact patients under complete program conditions; Fallers^UC100^: Number of fallers among 100 cognitively intact patients under usual care conditions.

## Competing interests

TPH is Director of Hospital Falls Prevention Solutions Pty Ltd. This is a research spin-off company that has been used to disseminate education of health professionals in the education program described in this manuscript. He has provided expert witness testimony to Minter Ellison Law Firm on the subject of the prevention of falls in hospitals. He has received payment to speak at conferences on the subject of the prevention of falls.

## Authors’ contributions

TPH contributed to study conception, design, trial management and undertaking analyses, as well as principal manuscript drafting, appraisal and editing. A-MH contributed to study conception, design, trial site management and data collection as well as manuscript appraisal and editing. KDH, SB, TH and CB contributed to study conception, design and manuscript appraisal and editing, SMMcP contributed to study conception, design, trial site management, data collection, analysis review as well as manuscript drafting, appraisal and editing. All authors read and approved the final manuscript.

## Pre-publication history

The pre-publication history for this paper can be accessed here:

http://www.biomedcentral.com/1741-7015/11/135/prepub
